# The impact of inflammatory response on psychological status of medical staff during COVID-19 pandemic

**DOI:** 10.1186/s41155-024-00335-w

**Published:** 2025-01-06

**Authors:** Dong Wang, Haijin Li, Yansong Liu, Hong Li, Yangyang Liu, Lijun Hou

**Affiliations:** 1 Department of Geriatric Psychiatry, Suzhou Mental Health Center, Suzhou Guangji Hospital, the Affiliated Guangji Hospital of Soochow University, Suzhou, China; 2 Department of Psychiatry, the First Affiliated Hospital of Zhengzhou University, Zhengzhou, China; 3 First People’s Hospital of Guannan County, Lianyungang, China; 4 The Affiliated Infectious Hospital of Soochow University, 10 Guangqian Road, Suzhou, Jiangsu 215131 China; 5 The Fifth People’s Hospital of Suzhou, Suzhou, China

**Keywords:** Psychological status, Inflammatory parameters, Psychopathology, SCL-90, COVID-19

## Abstract

**Background:**

Limited research has been conducted on the relationship between inflammatory markers and psychological status in medical staff fighting COVID-19.

**Objective:**

This article examines the psychological and inflammatory conditions of medical personnel working on the front lines of the battle against COVID-19. Methods: A total of 102 clinical staff members were included in this study. All subjects received the Symptom Checklist-90 questionnaire (SCL-90) and Posttraumatic Stress Disorder Checklist-Civilian questionnaires for assessing different mental symptoms. The levels of various inflammatory markers, including IL-1β, IL-2, IL-6, IL-8, TNF-a, and IFN-γ, along with GDNF, were evaluated.

**Results:**

Spearman correlation analysis showed that the levels of IL-6 were positively associated with the anxiety score (*Spearman’s rho* = .230, *p* = .021), obsessive–compulsive symptoms (*Spearman’s rho* = .201, *p* = .042). The levels of IL-8 were negatively associated with the anxiety score (*Spearman’s rho* = -.223, *p* = .028), obsessive–compulsive symptoms (Spearman’s rho = -.252, *p* = .012), hyperarousal (*Spearman’s rho* = -.221, *p* = .028). The levels of TNF-α were positively associated with the anxiety score (*Spearman’s rho* = .201, *p* = .045), obsessive–compulsive symptoms (*Spearman’s rho* = .222, *p* = .035).

**Conclusion:**

Generally, our results suggested that IL-6, IL-8 and TNF-α might play a role in the development of psychological symptoms among medical staff.

## Introduction

In December 2019, with the outbreak of Coronavirus disease 2019 (COVID-19) in Wuhan, Hubei, China, the world became affected by the spread of severe acute respiratory syndrome coronavirus 2 (SARS-CoV-2) (Verstrepen et al., 2020). Severe acute respiratory syndrome coronavirus 2 (SARS-CoV-2) is characterized by causing a highly contagious viral illness (Cascella et al., 2023). The widespread transmission of this virus presents a serious threat to public health. Due to lack of effective treatment and the high mortality rate in certain populations (Zhou et al., 2020a, 2020b), the actual pandemic has not only had severe physical implications for individuals, but has also posed a significant threat to mental well-being (Gao et al., 2020).

During previous high-stress and high-risk disease pandemics, medical staff often encountered huge mental burdens and various psychological barriers (Kang et al., 2018; Lehmann et al., 2015; Luo et al., 2020). Similarly, frontline medical staff remain vulnerable to infection and face a heightened risk of developing mental health issues amidst the ongoing COVID-19 pandemic (Dragioti et al., 2022; Zhou et al., 2020a, 2020b). A comprehensive review, encompassing a substantial dataset of prevalence meta-analyses, indicated that the prevalence of mental health problems, such as anxiety, depression, and acute stress symptoms, among hospital staff is generally elevated during the COVID-19 pandemic (Dragioti et al., 2022).

The occurrence of psychological disorders can trigger a cascade of pathways in the central nervous system, which subsequently activates stress responses in the autonomic nervous system (Liu et al., 2022). These responses can impact the process of inflammation (Marsland et al., 2007, 2017). A growing body of literature suggests that inflammatory processes play a role in the development of mental illnesses (Beurel et al., 2020; Müller, 2018). Previous research has shown that an inflammatory response can trigger mental health disorders, and chronic stress can exacerbate this response (Blanco-Melo et al., 2020). Meta-analyses have also provided evidence of a bidirectional relationship between depression and inflammation (Beurel et al., 2020; Smith, 1991). Liu et al. showed that psychological status, including depression, anxiety and stress was positively associated with levels of interleukin (IL)-6, while levels of IL-8 showed an inverse relationship (Liu et al., 2022). Earlier meta-analyses reported that IL-1β,IL-6, IL-10, and tumor necrosis factor (TNF)-α were related with moderation of psychological stress (Marsland et al., 2017). Evidence from these meta-analyses suggested three causal pathways: depression leading to inflammation, inflammation leading to depression, and bidirectional relationships. The immune and inflammatory reactions triggered by various stressors play a pivotal role in the pathogenesis of multiple psychiatric disorders such as depression, anxiety, and posttraumatic stress disorder (PTSD). Research conducted on both animal models and human subjects has progressively substantiated the notion that neuroinflammation contributes significantly to the multifaceted symptomatology observed in psychiatric illnesses. The pathological characteristics of immune responses vary depending on the nature of the stressor encountered, underscoring the intricate interplay between immunological processes and mental health manifestations (Doney et al., 2022; Zhao et al., 2019). Taking major depression as an example, animal research showed depression-like behavior can could be caused by IL-6 and IL-1 (Hammad et al., 2021; Liu et al., 2018; Smith, 1991). Positive associations have been found between IL-6, IL-1 and the severity of depression (Herbert & Cohen, 1993; Howren et al., 2009). In prospective studies, a higher level of depressive symptoms has been reported to associate with a higher level of IL-6 (Mac Giollabhui et al., 2021; Valkanova et al., 2013). Therefore, both the basic research and human studies mentioned above have all confirmed that inflammatory factors play a significant role in the pathogenesis of depression.

Despite previous studies that have demonstrated the potential correlation between various psychological symptoms and inflammatory factors, and the recognition of the importance of mental well-being among medical personnel in combating COVID-19 (Kang et al., 2020). There were limited studies exploring the connection between psychological status and inflammatory factors specifically in frontline medical staff during the COVID-19 pandemic. Therefore, the objective of this study is to examine the psychological and inflammatory statuses of both frontline and non-frontline medical staff engaged in the battle against the COVID-19 epidemic, and to assess the relationship between psychological profiles and the levels of different inflammation biomarkers, with the ultimate goal of providing improved mental health and psychosocial services to medical staff.

## Method

### Procedure

This study was approved by the Ethics Committee of Suzhou Guangji Hospital (No.2021020). The participants in the study all provided their informed consent for the test. To assess their psychological well-being, they were administered the Symptom Checklist-90 questionnaire (SCL-90) and the Posttraumatic Stress Disorder Checklist-Civilian. Additionally, blood samples were collected from the participants in order to conduct haemato-chemical tests.

### Participants

This study employed a cross-sectional research design and was conducted on 102 clinical staff engaged in the fight against COVID-19 at a hospital in Suzhou City, Jiangsu Province, from March 2020 to March 2022, using the convenience sampling method. The participants were divided into two groups based on the inclusion criteria for front-line medical staff, which included doctors and nurses in direct contact with patients with COVID-19 (Wang et al., 2022). Forty-nine healthcare professionals were designed as non-front-line group and 53 were classified as front-line clinical personnel. Exclusion criteria included any previous neurological, psychiatric, or serious systemic illnesses, as well as drug abuse. Moreover, we eliminated entries with invalid or incomplete scales, inadequate patient profiles, or records lacking patient consent.

For evaluation and examination, we utilized online system questionnaires and self-assessment tools. The participants voluntarily completed the forms and assessment scales. To ensure data reliability, we meticulously collected and observed it via the questionnaire backend. Furthermore, two researchers conducted thorough evaluations of the extracted data to maintain its integrity.

### Measures

#### The Symptom Checklist 90

The SCL-90 is a multidimensional symptom self-report inventory consisting of 90 items and nine factor scores with sufficient reliability and validity. Each item received a score on a five-point scale from “not-at all” to “extremely”. The nine factor scores included average scores for positive items, somatization, obsessive–compulsive symptoms (OCS), interpersonal sensitivity, depression, anxiety, hostility, terror, paranoia ideation, and psychosis (Derogatis et al., 1973, 1976; Holi et al., 1998).

#### PTSD Checklist-Civilian Version

The PTSD Checklist-Civilian Version (PCL-C) (Conybeare et al., 2012) was used to measure post-traumatic stress symptoms among medical staff against COVID-19. The response options ranged from one (not at all) to five (seriously). The reliability and validity of this questionnaire have been previously assessed (Zhang et al., 2011a). It consists of 17 items, and the total scores range from 17 to 85. Participants with a cut-off score of 38 or higher were classified as having PTSD symptoms (Grubaugh et al., 2007; Zhang et al., 2011). In order to investigate the reproducibility and consistency of SCL-90 and PCL-C, reliability coefficients as measured by Cronbach’s alpha were calculated.

#### Measurement of biological parameters

For the measurement of inflammatory markers and glial cell line-derived neurotrophic factor (GDNF), we employed an enzyme immunoassay using a commercially available kit (Boster, Wuhan, China). We conducted the measurements in duplicate, following the manufacturer’s instructions. The levels of various inflammatory markers, including IL-1β, IL-2, IL-6, IL-8, TNF-a, and IFN-γ, along with GDNF, were evaluated in a manner that guaranteed the anonymity of the samples. The sensitivities for the experiment were as follows: IL-1β was measured at a concentration of 0.15 pg/ml. IL-8, GDNF, TNF-α, IFN-γ, and IL-2 were measured at a concentration of 1 pg/ml. IL-6 was measured at a concentration of 0.3 pg/ml. The inter-assay and intra-assay coefficient of variation values were found to be below 6.8% and 6.3% respectively.

### Statistical analysis

The statistical analysis was performed using IBM SPSS Statistics 25.0. The normality of the data was analyzed by the Shapiro–wilk test. Non-normally distributed data are expressed as median (interquartile range [IQR]), while normally distributed data as mean ± standard deviation (M ± SD). Chi-squared and Fisher’s tests were used to compare proportions such as gender, education, marital status, and comorbidities. Group differences in nonparametric data including psychometric scale scores and inflammatory factor levels were compared by the Kruskal–Wallis test. The Cronbach’s α coefficient was employed to evaluate the internal consistency of the items in SCL-90 and PCL-C. We chose Spearman correlation analysis to explore the relationship between psychometric scale results that were determined to be significantly different and inflammatory factors. After controlling for demographic factors, we used SCL-90 among COVID-19 medical staff as the dependent variable. Age, marital status, and inflammatory factors served as independent variables. We performed stepwise multiple linear regression analyses to evaluate the impact of serum inflammatory markers on mental status.

## Results

Cronbach’s alpha coefficients were computed to evaluate the reliability of individual symptomatic measures within the SCL-90 and PCL-C assessments. Both measures exhibited strong internal consistency, with values exceeding 0.70 across all dimensions. Specifically, the overall Cronbach’s α for the SCL-90 survey was 0.83, varying from 0.81 for phobic anxiety to 0.95 for psychoticism. The overall Cronbach’s α for the PCL-C was 0.85, ranging from 0.82 for avoidance to 0.93 for re-experience. These data confirm satisfactory reliability across the board.

The demographic and clinical characteristics of both frontline and non-frontline medical staff during the COVID-19 epidemic are presented in Table 1. A total of 102 medical staff members were included in the survey. The mean age of frontline medical staff (33 years, range 31–39) was found to be higher compared to non-frontline staff (31 years, range 26.5–35.5). This difference between the two groups was statistically significant (*p* = 0.003). There was also a statistically significant difference in marital status (*p* = 0.008). However, no significant differences appeared between the two groups in terms of gender, education level, body mass index (BMI), and physical comorbidities.

**Table 1:** Socio-demographic and clinical analysis between frontline and non-frontline medical staff under the COVID-19 epidemic

Variables M (P_25_, P_75_)	Frontline medical staff	Non-frontline medical staff	*Z/X*^*2*^	*p*
**Age, years**	33(31,39)	31(26.5,35.5)	-2.9	.003
**Gender**			1.89	.168
Male	18.9%	81.1%		
Female	30.6%	69.4%		
**Education**			3.48	.289
junior high school *n* (%)	9.4%	4.1%		
High school, *n* (%)	81.81%	79.6%		
University degree, *n* (%)	7.5%	16.3%		
Master’s degree, *n* (%)	1.9%	0%		
**Marital status**			8.50	.008
Single, *n* (%)	18.9%	44.9%		
Married, *n* (%)	79.2%	55.1%		
Widowed or divorced *n* (%)	1.9%	0%		
**Comorbidities**			0.38	.535
Yes	18.9%	14.3%		
No	81.1%	85.7%		
**BMI, kg/m2**	22.83(21.45,26.70)	23.12(21.12,27.34)	-0.07	.941

*Abbreviations*: *BMI* Body mass index, *M (P25, P75)* Median (25th percentile, 75th percentile)

After adjusting for age, gender, education, and marital status, our study revealed that first-line medical staff exhibited higher scores in OCS (*p* < 0.001), depression (*p* = 0.007), anxiety (*p* = 0.003), PCL-sum (*p* = 0.011), re-experience (*p* = 0.028), and hyperarousal (*p* = 0.001), as indicated in Table 2. Subsequently, we compared the levels of serum inflammatory factors. The findings, depicted in Table 3, demonstrated that the frontline medical staff group displayed significantly elevated levels of IL-1β (*p* = 0.001) and TNF-α (*p* < 0.001). In contrast, non-frontline medical staff showed a significant increase in the inflammatory markers IL-8.

**Table 2:** Psychometric scale differences between frontline and non-frontline medical staff under the COVID-19 epidemic

Variables	Frontline medical Staff, M (P_25_, P_75_)	Non-frontline medical staff, M (P_25_, P_75_)	*Z*	*p*
**PCL-sum**	29.00(24.25,34.00)	26.00(19.50,32.00)	-2.53	.011*
re-experience	29.50(24.00,34.00)	26.00(19.5,32)	-2.19	.028*
Avoidance	7.00(5.00,8.75)	6.00(4.00,10.00)	-0.37	.710
hyperarousal	12.00(10.00,14.75)	10.00 (8.00,12.00)	-3.22	.001***
**SCL-90**	127.5 0(103.25,153.50)	111.00 (105.00,126.00)	-2.52	.012*
Somatization	14.50 (12.00,20.00)	13.00 (12.00,16.00)	-1.82	.068
OCS	18.00 (14.00,23.50)	13.00 (11.00,16.00)	-3.72	.001***
Interpersonal sensitivity	13.00 (10.00,17.00)	13.00 (10.00,14.50)	-1.02	.311
Depression	19.00 (14.00,25.00)	15.00 (13.00,20.50)	-2.68	.007**
Anxiety	13.50 (11.00,18.00)	12.00 (10.50,13.50)	-2.97	.003**
Hostility	8.00 (7.00,10.00)	7.00 (6.00,10.00)	-1.47	.141
Phobic anxiety	8.50 (7.00,11.00)	8.00 (7.00,9.00)	-1.59	.112
Paranoid ideation	8.00 (6.00,10.75)	8.00 (6.00,9.00)	-1.21	.232
Psychoticism	12.00 (10.00,14.00)	11.00 (10.00,13.00)	-1.26	.214
Other	10.00 (9.00,12.00)	10.00 (9.00,11.00)	-0.35	.720

*Abbreviations*: *PCL-C* Posttraumatic stress disorder (PTSD) Checklist-Civilian, *OCS*: Obsessive–compulsive symptoms, *M (P25, P75)* Median (25th percentile, 75th percentile)

^*^p < .05, **p < .01, *** *p* < .001

**Table 3:** Comparison of laboratory parameters between frontline and non-frontline medical staff under the COVID-19 epidemic

Variables	Frontline medical staff, M (P_25_, P_75_)	Non-frontline medical staff, M, (P_25_, P_75_)	*Z*	*p*
IL-1β	2.75(2.12,3.76)	1.49(0.85,3.56)	-3.38	.001***
IL-2	4.27(3.05,6.10)	3.51(2.26,5.15)	-1.50	.132
IL-6	2.96(2.40,3.45)	2.72(2.24,3.15)	-1.48	.141
IL-8	7.98(7.72,8.55)	9.12(8.89,9.95)	-7.34	.001***
TNF-α	2.23(0.60,6.26)	0.60(0.19,1.85)	-4.09	.001***
IFN-γ	2.42(0.54,4.31)	1.84(1.41,2.69)	-0.38	.701
GDNF	12.33(1.34,13.73)	11.76(11.15,12.37)	-1.48	.143

*Abbreviations*: *IL* Interleukin, *TNF-α* Antitumor necrosis factor-α, *IFN-γ* Interferon-γ, *GDNF* Glial cell line-derived neurotrophic factor, *M (P25, P75)* Median (25th percentile, 75th percentile)

^*^p < .05, **p < .01, *** *p* < .001

The correlation analysis between inflammatory markers and psychological symptoms in all individuals is presented in Table 4. The levels of IL-6 exhibited a significant positive association with the anxiety score (*Spearman’s rho* = 0.230, *p* = 0.021) and OCS (*Spearman’s rho* = 0.201, *p* = 0.042). Conversely, the levels of IL-8 displayed a negative association with the anxiety score (*Spearman’s rho* = -0.223, *p* = 0.028), OCS (*Spearman’s rho* = -0.252, *p* = 0.012), and hyperarousal (*Spearman’s rho* =-0.221, *p* = 0.028). Additionally, the levels of TNF-α showed a significant positive association with the anxiety score (*Spearman’s rho* = 0.201, *p* = 0.045) and OCS (*Spearman’s rho* = 0.222, *p* = 0.035). After adjusting for demographic factors, results from linear regression indicated that the inflammatory factor IL-6 had a significant impact on SCL-90 scores (*R*^*2*^ = 0.284, *F* = 37.461,* t* = 2.533, *β* = 0.212, *p* = 0.013).

**Table 4:** Spearman correlation analysis between psychometric scale results and inflammatory markers

Variables	Anxiety	depression	OCS	SCL-90	PCL	Re-experience	hyperarousal
IL-1β	.064	.079	.071	.024	-.004	-.019	-.002
IL-2	.037	.066	.036	.953	-.057	-.046	.013
IL-6	.230*	.181	.201*	.064	.450	.691	.601
IL-8	-.223*	-.142	-.252*	-.151	-.181	-.143	-.221*
TNF-α	.201*	.133	.222*	.086	.222*	.233*	.263*
IFN-γ	.007	-.001	.004	-.029	-.018	.087	-.059
GDNF	.017	.200*	.035	.522	.134	.007	.015

*Abbreviations*: *IL* Interleukin, *TNF-α* Antitumor necrosis factor-α, *IFN-γ* Interferon-γ, *GDNF* Glial cell line-derived neurotrophic factor, *OCS* Obsessive–compulsive symptoms, *PCL* Posttraumatic Stress Disorder (PTSD) Checklist-Civilian

^*^p < .05, **p < .01, *** *p* < .001

## Discussion

Despite the substantial body of evidence regarding the mental well-being of medical staff during the COVID-19 pandemic, limited research exists on the relationship between mental status and inflammation markers for both frontline and non-frontline medical staff. In this study, we aimed to investigate the connection between psychological status and serum levels of inflammatory parameters in medical staff working against COVID-19. Our findings indicate that psychological status, including anxiety and OCS, correlates with levels of IL-6 and IL-8, even after adjusting for confounding factors. Additionally, we identified other factors that correlate with scores on psychological test items. These results suggest that inflammatory factors may impact the mental well-being of medical staff in their fight against COVID-19. Furthermore, inflammation may play a role in the development of psychological disorders and certain affective symptoms.

Proinflammatory cytokines play a significant role in the normal functioning of synaptic and neural plasticity. However, when their levels increase, they can adversely affect cellular processes, leading to neuronal loss, neuronal atrophy, and altered gene expression (Tang et al., 2018). Among these cytokines, IL-6 is particularly important as it mediates T cell and B cell activation and has disease-promoting inflammatory effects (Akdis et al., 2016; Raison et al., 2018). IL-6 has been extensively studied and can potentially influence neurotransmission (Costello et al., 2019). In our study, we observed elevated levels of IL-6 and anxiety scores in first-line medical staff compared to non-first line medical staff, consistent with previous research. For instance, a meta-analysis revealed that IL-6 commonly increased in individuals with anxiety disorders compared to controls in most studies (Costello et al., 2019). Additionally, a large cohort study demonstrated significantly higher levels of IL-6 in individuals with generalized anxiety disorder (GAD) (Vogelzangs et al., 2013). However, our findings also showed a positive association between IL-6 and anxiety, which contradicts the results of some previous studies. Notably, two studies exploring the relationship between anxiety and inflammation found no association between IL-6 and anxiety symptoms (Milaneschi et al., 2021; Vogelzangs, et al., 2013). This inconsistency in results may stem from various confounding factors, including participant heterogeneity, sample size, and differences in parameters such as the scales used.

The first-line medical staff exhibited higher levels of IL-6 and OCS scores. Moreover, a correlation between IL-6 and OCS scores was observed. This finding aligns with previous studies to some extent. For instance, Dania Jose et al. discovered elevated IL-6 levels in obsessive–compulsive disorder (OCD) patients. However, they concluded that plasma IL-6 levels did not correlate with the severity of the illness as measured by the Yale-Brown Obsessive Compulsive Scale (YBOCS) (Jose et al., 2021). This indicate that heightened IL-6 may serve as an inflammatory marker for OCS. Furthermore, a meta-analysis indicated a potential association between immune dysregulation and OCD, although it remains unproven (Cosco et al., 2019). Therefore, further research is needed. Additionally, age has been pointed as a critical factor, as people age, rates of depression and inflammation tend to increase(Alexopoulos & Morimoto, 2011a; Blazer, 2003; Cohen et al., 1997). Thus, further investigation is warranted. Therefore, we controlled for the effect of age and identified an association between SCL-90 and IL-6. This finding suggests a potential causal role of IL-6 in affective symptoms.

IL-8 is a chemokine that plays a dual role in both the immune system and neuroprotection. The association between IL-8 and anxiety has been the subject of limited research, making it difficult to draw definitive conclusions. Our results showed that IL-8 levels were higher in non-firstline medical staff and were negatively correlated with anxiety. A study conducted on a large cohort of patients provided evidence of a correlation between low levels of IL-8 and increased psychiatric symptoms of anxiety (Janelidze et al., 2015). These results suggest that decreased IL-8 levels might contribute to the pathophysiological changes associated with anxiety symptoms, particularly in patients displaying such symptoms (Janelidze et al., 2015). Additionally, a decrease in serum IL-8 levels has been observed in individuals with PTSD, a prevalent stress-related mental disorder (Song et al., 2007). However, a study about selective serotonin reuptake inhibitors (SSRIs) showed baseline levels of anxiety and pro-inflammatory cytokines including IL-8 were significantly reduced after the treatment of SSRIs (Hou et al., 2019). Interestingly, the elevated IL-8 levels observed in non-firstline medical staff do not align with previous findings, which reported significantly higher IL-8 levels in patients with GAD (Janelidze et al., 2015; Tang et al., 2018). Collectively, these findings suggest that IL-8 might be a causal risk factor for anxiety.

TNF-α, a notable inflammatory mediator primarily synthesized by macrophages, holds significant importance as a signaling molecule in inflammation and apoptosis (Goodsell, 2006). The elevation of TNF-α levels has proven to correlate with a decrease in neuronal synaptic plasticity, neurotropic factors, and neurogenesis (Hashmi et al., 2013). In the light of the evidence of an experimental study, TNF-α can increase the permeability of the blood brain barrier, facilitating the entry of other cytokines and immune cells into the brain, thereby contributing to the manifestation of psychological symptoms (Cheng et al., 2018; Varatharaj & Galea, 2017). At a molecular level, TNF can reduce the availability of monoamines which has been suggested to link with the mechanism of anxiety (Miller & Raison, 2016). Our results showed that TNF-a was positively associated with anxiety, consistent with previous research findings. Moreover, proinflammatory cytokines like TNF-α have been implicated in triggering anxiogenic responses in adult animals (Connor et al., 1998; Koo & Duman, 2009). Overall, our results suggest that IL-6, IL-8, and TNF-α may play a role in the pathogenesis of psychological disorders. However, larger and longitudinal studies are required to validate our findings.

### Limitation

The study encountered several limitations that need to be acknowledged. Firstly, the limited sample size, the weak correlation identified, the diverse composition of the sample, and the differences observed between online and traditional in-person surveys, could potentially impact the broad applicability of the results. Secondly, the cross-sectional design of the study prevented us from establishing causal relationships between psychological status and inflammatory biomarkers. Thirdly, the study did not account for potential interactions between emotional states like depression and anxiety, highlighting the need for further investigation. Fourthly, it is worth noting that all diagnoses in this study pertained to mental health states and not mental disorders. Lastly, the significance of subclinical infectious parameters, gender, alcohol, tobacco, and drug consumption in analyzing trends in an open population cannot be overlooked. Limited data on these factors may impact inflammation variability, urging careful interpretation of findings for a more holistic understanding.

## Conclusion

In this study, our findings indicate that IL-6, IL-8, and TNF-α are potentially involved in developing psychological symptoms. Nonetheless, uncertainty remains regarding the causal relationship between inflammation and psychological disorders. It is important to note that our results contribute to the growing body of evidence supporting the idea that inflammatory factors may have a symptom-specific effect. These findings enhance our comprehension of the intricate connection between inflammation and psychological well-being.

## Data Availability

The data underlying this article will be shared on reasonable request to the corresponding author.

## Abbreviations

COVID-19: Coronavirus disease 2019
IL: Interleukin
TNF: Tumor necrosis factor
SCL-90: Symptom Checklist-90 questionnaire
PTSD: Posttraumatic Stress Disorder
PCL-C: PTSD Checklist-Civilian Version (PCL-C)
GDNF: Glial cell line-derived neurotrophic factor
IQR: Interquartile range
M ± SD: Mean ± standard deviation
BMI: Body mass index
GAD: Generalized Anxiety Disorder
OCS: Obsessive-compulsive symptoms
